# Screening of the key response component groups and mechanism verification of Huangqi-Guizhi-Wuwu-Decoction in treating rheumatoid arthritis based on a novel computational pharmacological model

**DOI:** 10.1186/s12906-023-04315-y

**Published:** 2024-01-02

**Authors:** Qinwen Liu, Qian Luo, Qiling Fan, Yi Li, Aiping Lu, Daogang Guan

**Affiliations:** 1 Department of Biochemistry and Molecular Biology, School of Basic Medical Sciences, Southern Medical University, Guangzhou, China; 2 Guangdong Provincial Key Laboratory of Single Cell Technology and Application, Southern Medical University, Guangzhou, China; 3 Institute of Integrated Bioinformedicine and Translational Science, Hong Kong Baptist University, Hong Kong, China; 4 Guangdong-Hong Kong-Macau Joint Lab on Chinese Medicine and Immune Disease Research, Guangzhou, China

**Keywords:** Rheumatoid arthritis, Huangqi-Guizhi-Wuwu-Decoction, Network pharmacology, Key response components group

## Abstract

**Background:**

Rheumatoid arthritis (RA) is a chronic autoimmune disease characterized by the destruction of synovial tissue and articular cartilage. Huangqi-Guizhi-Wuwu-Decoction (HGWD), a formula of Traditional Chinese Medicine (TCM), has shown promising clinical efficacy in the treatment of RA. However, the synergistic effects of key response components group (KRCG) in the treatment of RA have not been well studied.

**Methods:**

The components and potential targets of HGWD were extracted from published databases. A novel node influence calculation model that considers both the node control force and node bridging force was designed to construct the core response space (CRS) and obtain key effector proteins. An increasing coverage coefficient (ICC) model was employed to select the KRCG. The effectiveness and potential mechanism of action of KRCG were confirmed using CCK-8, qPCR, and western blotting.

**Results:**

A total of 796 key effector proteins were identified in CRS. The Kyoto Encyclopedia of Genes and Genomes (KEGG) and Gene Ontology (GO) analyses confirmed their effectiveness and reliability. In addition, 59 components were defined as KRCG, which contributed to 85.05% of the target coverage of effective proteins. Of these, 677 targets were considered key reaction proteins, and their enriched KEGG pathways accounted for 84.89% of the pathogenic genes and 87.94% of the target genes. Finally, four components (moupinamide, 6-Paradol, hydrocinnamic acid, and protocatechuic acid) were shown to inhibit the inflammatory response in RA by synergistically targeting the cAMP, PI3K-Akt, and HIF-1α pathways.

**Conclusions:**

We have introduced a novel model that aims to optimize and analyze the mechanisms behind herbal formulas. The model revealed the KRCG of HGWD for the treatment of RA and proposed that KRCG inhibits the inflammatory response by synergistically targeting cAMP, PI3K-Akt, and HIF-1α pathways. Overall, the novel model is plausible and reliable, offering a valuable reference for the secondary development of herbal formulas.

**Supplementary Information:**

The online version contains supplementary material available at 10.1186/s12906-023-04315-y.

## Introduction

Rheumatoid arthritis (RA) is a common chronic inflammatory disease, with a prevalence ranging from 0.4% to 1.3% depending on factors such as sex, age, and region [1–3]. Current clinical treatments for RA include non-steroidal anti-inflammatory drugs (NSAIDs), disease-modifying antirheumatic drugs (DMARDs), glucocorticoids, and other biological agents. However, these drugs have side effects that limit their long-term use [1, 4–6]. Traditional Chinese medicine (TCM) has been used for centuries to treat complex diseases with minimal side effects [7–9]. One TCM formula, Huangqi-Guizhi-Wuwu-Decoction (HGWD), has shown significant anti-inflammatory effects in the treatment of RA. However, the specific components and mechanisms of action of HGWD in RA treatment require further investigation.

HGWD consists of five botanicals: *Astragalus mongholicus* Bunge. (Huang Qi, HQ, 15 g), *Neolitsea cassia (L.)* Kosterm*.* (Gui Zhi, GZ, 12 g), *Paeonia lactiflora* Pall. (Shao Yao, SY, 12 g), *Zingiber officinale* Roscoe. (Sheng Jiang, SJ, 25 g) and *Ziziphus jujuba* Mill. (Da Zao, DZ, 4). This combination reflects the characteristic complexity of TCM formulas, which involve multiple components, pathways, links, and targets. The complexity of TCM formulas poses challenges for studying their pharmacological mechanisms. Network pharmacology, a new discipline, offers a research strategy that encompasses the comprehensive, systematic, and dynamic nature of TCM, thus aiding in the modernization of TCM. Our previous work has successfully decoded the pharmacological mechanisms of various TCM formulas in various diseases, including liver cancer, osteoporosis, systemic lupus erythematosus, and RA [10–13].

The key response component group (KRCG) and its mediated core functional space (CFS) are the active components and targets tightly associated with disease progression in TCM formulas. They are also a key part of exerting efficacy in the entire prescription and a key clue to exploring pharmacological mechanisms. In this study, we explored the KRCG and CFS of HGWD in treating RA and validated their effectiveness at the functional level using Kyoto Encyclopedia of Genes and Genomes (KEGG) and Gene Ontology (GO) analyses. Furthermore, we selected four components (moupinamide, 6-Paradol, hydrocinnamic acid, and protocatechuic acid) for in vivo validation, and the results demonstrated their effectiveness in inhibiting RA inflammatory responses. Western blotting experiments further revealed their synergistic effects through multiple pathways and targets.

## Analytical methods

### Chemical components and potential targets collection

The ingredients of HGWD were collected from four online databases: the Traditional Chinese Medicine Systems Pharmacology (TCMSP) database [14], Traditional Chinese Medicine integrated database (TCMID) [15] and Traditional Chinese Medicine database@Taiwan [16]. Potential targets were obtained using several predictive tools, including the Similarity Ensemble Approach SEA (https://sea.bkslab.org/) [17], HitPick (http://mips.helmholtz-muenchen.de/proj/hitpick) [18], and Swiss Target Prediction (http://www.swisstargetprediction.ch/) [19]. The data were duplicated and integrated.

### Active chemical components identification

ADME properties refer to human absorption, distribution, metabolism, and excretion of exogenous chemicals, which are key characteristics for evaluating whether small-molecule drugs can be used as new drugs. This is a requirement of regulatory agencies worldwide. In this study, two ADME characteristics, drug-like bioavailability (DL) and oral bioavailability (OB), were used to screen chemical components with potential biological activity. Based on this, compounds with DL ≥ 0.18, and OB ≥ 30% were selected, and a further sum combined with the screening criteria of MW < 500 Da, Rotatable Bond Count < 10, Hydrogen Bond Acceptor Count < 10, Hydrogen Bond Donor Count < 5, 20 ≤ TPSA ≤ 130, -0.7 ≤ XLogP3 ≤ 5.0, -0.4 ≤ WLogP ≤ 5.6, MLogP ≤ 4.15, ESOL Class, Ali Class and Silicos-IT class except poorly soluble, hERG inhibition except high risk, and GI absorption retaining high absorption, the remaining compounds were identified as potential active ingredients.

### Construct the CFS and evaluate the effective proteins

TCM usually plays a therapeutic role in the form of multi-component-target pathways in complex diseases. These components and their targets coexist to orchestrate a potential action network. Determining the CFS from this complicated network can retain the components, targets, and pathogenic genes that are highly related to prescription function to the greatest extent. We mapped the component-target-disease-pathogenic gene relation to the PPI network, which was composed of the data compiled by the online tools CMGRN and PTHGRN and the data from the BioGRID database. The influence of the network nodes is the foundation for screening the key components of a network. Many methods have been proposed for calculating the influence of nodes, such as betweenness centrality, proximity centrality, clustering coefficient, and the average shortest path length. In this study, we developed a novel methodological program to determine the influence of nodes. ${\text{CW}}{}_{ctp}=\left\{C,W\right\}$ is defined as the entire C-T-P network, *C* represents the ingredients, target genes, or pathogenic genes. *W* represent ingredients-target-pathogenic gene interactions:

$$\aleph =\underset{i<n}{{\text{max}}}({t}_{1\to 2},{t}_{1\to 3},{t}_{1\to 4}\cdots {t}_{i\to s}\cdots {t}_{(\frac{n\left(n-1\right)}{2}-1)\to \frac{n\left(n-1\right)}{2}})$$

$${IS}_{e}=\sqrt{\frac{\left(T+1\right)-\sum {g}_{sv}(e)/m}{\aleph }\times \sum_{s,t\in V}\frac{{g}_{SV}(e)}{{g}_{sv}}}$$

$$I{S}_{median}=median\left\{{IS}_{1}, {IS}_{2},{IS}_{3},\cdots ,{IS}_{n}\right\}$$

$${\text{CFS}}=\bigcup_{i=1}^{n}{IS}_{\left(NW{}_{ctp}\right)i}>{IS}_{median}$$

${IS}_{e}$: Influence of node e in the NW

$\aleph$: represents the diameter of the CW network, that is, the distance between the two farthest nodes in the network. If the network is discontinuous, then $\aleph$ represents the diameter of the largest connected network.

${g}_{sv}$: count of path-link nodes s and v

Here, ${g}_{sv}(e)$: represents the total number of paths connecting nodes s and v through node e and m represents the total number of shortest paths passing through node e in the CW network. where n indicates the total number of ingredients, target genes or pathogenic genes in the CW network.

### The increasing coverage coefficient (ICC) model for determining KRCG

To better determine KRCG from CFS, we design an increasing coverage coefficient model. In this model, ${w}_{i}$ denotes the network occupation rate of component i in CFS, ${v}_{i}$ denotes the occupation rate of target genes for pathogenic genes, and the maximum expected network occupation rate of KRCG is R. $R>0, {w}_{i}>0, {v}_{i}>0, 1\le i\le n$. The design formula is as follows:

$$\mathit{ICC}=\underset{}{{\text{max}}}\sum_{i=1}^{n}{v}_{i}{x}_{i}$$

$$\sum_{i=1}^{n}{w}_{i}{x}_{i}\le R {x}_{i}\in \left\{\mathrm{0,1}\right\}, 1\le i\le n$$

The sub-problems of the question can be solved as:

$${\mathit{ICC}}_{\mathit{sub}}=\underset{}{{\text{max}}}\sum_{k=1}^{n}{v}_{k}{x}_{k}$$

$$\sum_{k=1}^{n}{w}_{k}{x}_{k}\le {R}_{sub} {x}_{k}\in \left\{\mathrm{0,1}\right\}, 1\le k\le n$$

When the expected network occupation rate is ${R}_{sub}$ and the optimized component is y, ${\text{m}}({\text{i}}, {C}_{sub})$ is the optimized solution. According to the properties of the optimized substructure, the recursive process for validating ${\text{m}}({\text{i}}, {C}_{sub})$ can be described by the following formula:

$$m\left(i, {R}_{sub}\right)=\left\{\begin{array}{c}max\left\{m\left(i+1, {R}_{sub}\right), m\left(i+1, {R}_{sub}-{w}_{i}\right)+{v}_{i}\right\} {R}_{sub}\ge {w}_{i}\\ m\left(i+1, {R}_{sub}\right) 0\le {R}_{sub}<{w}_{i}\end{array}\right.$$

$$m\left(n, {R}_{sub}\right)=\left\{\begin{array}{c}{v}_{n} {R}_{sub}\ge {w}_{n}\\ 0 0\le j<{w}_{n}\end{array}\right.$$

### Function enrichment analysis

The ClusterProfiler package of R (version 4.2.2) was used to perform Gene Ontology (GO) and Kyoto Encyclopedia of Genes and Genomes (KEGG) pathway analysis with an adjusted *p* value cutoff of 0.05 and a q-value of 0.05. Graphs were created using the ggplot2 package in R software (version 4.2.2). Data visualization was performed using Pathview in the R Bioconductor package (https://www.bioconductor.org/) and Cytoscape [20].

## Experimental verification

### Materials

Moupinamide (CAS:66648–43-9), 6-Paradol (CAS:27113–22-0), hydrocinnamic acid (CAS:501–52-0), and protocatechuic acid (99–50-3) were obtained from Jingzhu Biotechnology (Nanjing, China). Lipopolysaccharide (LPS) was obtained from Sigma-Aldrich (St. Louis, MOUSA).

### Cell culture

RAW264.7 cells were cultured in DMEM (Invitrogen, Shanghai, China) supplemented with 10% FBS (Life Technologies), 100 μg/mL streptomycin, and 100 μg/mL penicillin, and incubated at 37 °C under 5% CO_2_.

### Cell viability assay

The cells were seeded in 96-well plates at a density of 1 × 10^4^ cells/well. On the second day, different concentrations of drugs were added and incubated for 24 h. Then, 10 μL of the CCK-8 reagent was added for 2 h at a constant temperature. Finally, the results were quantified by recording the absorbance of the solution was measured at 450 nm.

### Measurement of NO content

RAW264.7 was inoculated in 12-well plates and pre-protected with 1 μg/ml LPS for 2 h after adherent, followed by intervention with a specified concentration component. After 24 h, the supernatant of the cell culture was collected, and the NO content was measured according to the instructions of the Total Nitric Oxide Assay Kit (Beyotime, China).

### Quantitative real-time PCR (qRT-PCR)

Total RNA was extracted using a Total RNA Isolation Reagent Kit (Qiagen, China). RNA was reverse-transcribed into cDNA using the TransScript® Uni All-in-One First-Strand cDNA Synthesis SuperMix for qPCR kit (TransGen Biotech, China) and quantitative PCR was performed using SYBR Premix (Vazyme Biotech Co., Ltd.). All experiments were performed using QuantStudio 1.

### Western blot

Total protein was extracted using RIPA lysis buffer. Equal amounts of protein samples (20–30 μg) were separated using SDS-PAGE and transferred onto polyvinylidene difluoride (PVDF) membranes. Membranes were first blocked with 5% skim milk for 1 h and then immunoblotted with primary antibodies at 4 °C overnight. The membranes were washed with TBST three times for 5 min each and then incubated with a secondary antibody at room temperature for 1 h. Finally, the membranes were washed again, as described above. Antibody signals were detected using an electrochemiluminescence (ECL) substrate. All antibodies were purchased from CST: β-actin (1:1,000; cat. no. #13E5), HIF-1α (cat. no. 36169 T) and PI3K (cat. no. 4257 T), Phospho-PI3K (cat. no. 4228 T), AKT (Cat. no. 4691 T) and phospho-AKT (cat. no. 4060 T), PKA C-α (cat. no. 5842 T) and phospho-PKA C (cat. no. 5661 T).

### Statistical analysis

Data are expressed as the mean SD. All statistical analyses were performed using GraphPad Prism 7.0. Statistical significance was set at * 0.01 < *P* < 0.05, ** 0.001 < *P* < 0.01, and *** *P* < 0.001.

## Results

### Selection and identification of RA-related pathogenic genes

**Fig. 1:**
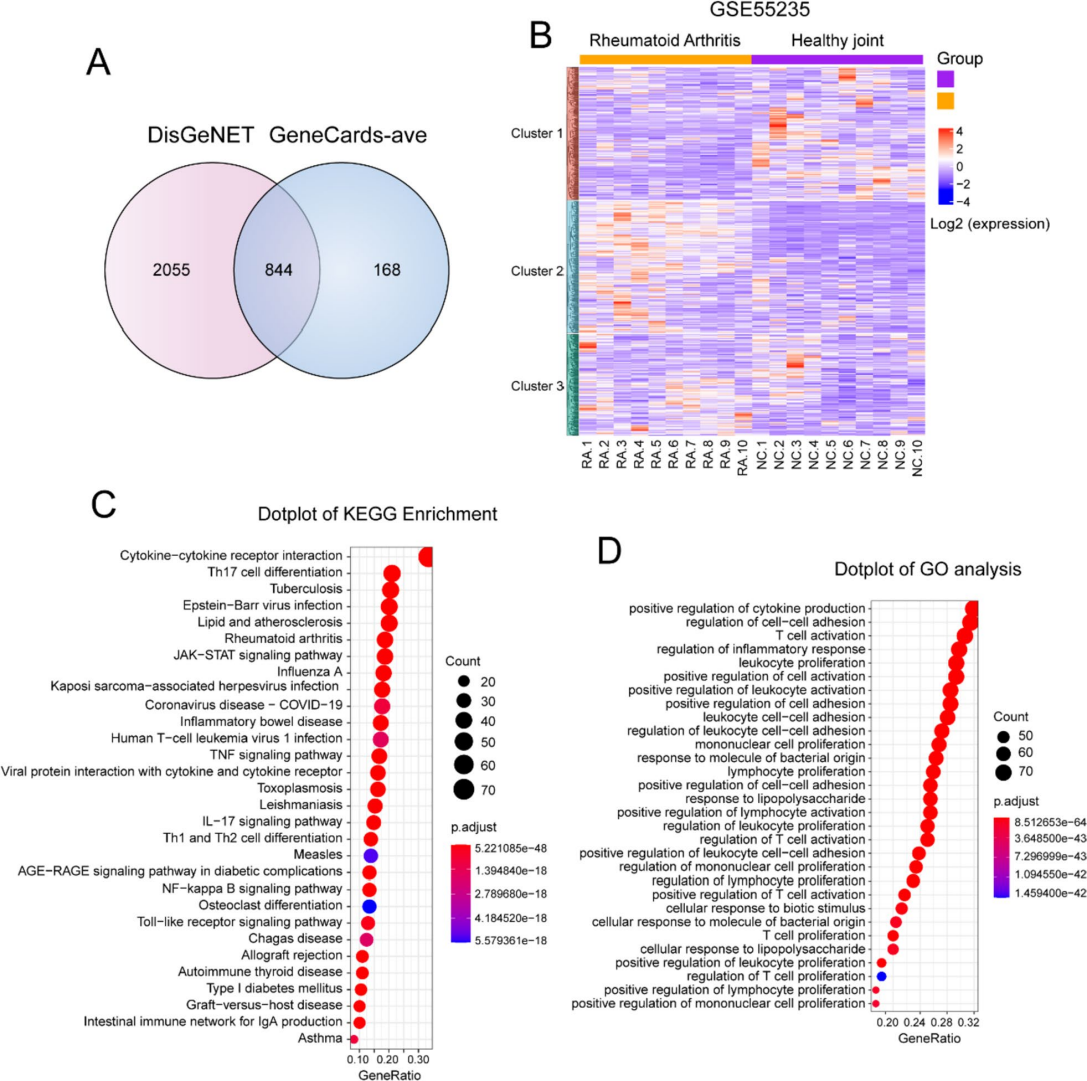
Selection and identification of RA-related pathogenic genes. **A** The venn diagram of overlapping genes between DisGeNET and GeneCards-ave. **B** The gene expression heatmap of the RA-related pathogenic genes between RA and negative control (NC). **C**-**D** The top 30 KEGG pathways and GO terms of RA-related pathogenic genes

Potential RA-related pathogenic genes were identified using the GeneCards and DisGeNET databases. 1012 pathogenic genes with “relevance scores” greater than the average of pathogenic genes from GeneCards (GeneCards-ave) were selected to overlap with the pathogenic genes from DisGeNET. A total of 844 shared pathogenic genes were figured out as RA-related (Fig. 1A, Supplementary Table S1, sheet 1–3). To verify whether these pathogenic genes were highly correlated with RA, we extracted the expression matrix of these pathogenic genes from the GEO database (Series: GSE55235) and performed a heat map analysis. The results confirmed that these pathogenic genes could distinguish RA from healthy samples and accurately reflect the changes in pathological genes in the pathogenesis of RA (Fig. 1B). KEGG pathway and GO term analyses are important for revealing the functions of target genes. As shown in Fig. 1C, these gene-enriched pathways, including the rheumatoid arthritis pathway, NF-κB pathway, TNF pathway, and osteoclast differentiation pathway, are well documented in the pathogenesis of RA. Furthermore, GO analysis of molecular functions showed that these pathogenic genes were related to the production of various cytokines and activation of multiple immune cells (Fig. 1D). Additionally, the top ten ranked pathogenic genes with the highest number of evidences were closely related to inflammation. For example, TNF-α, the core cytokine in the inflammatory cascade, has been used as a target for RA treatment with excellent efficacy [21, 22]. The HLA-DRB1 allele constitutes the strongest genetic association for RA and may account for at least 30% of the total genetic component [23, 24]. Moreover, IL-1β, IL-17A, and IL-10 levels strongly correlated with RA progression of RA [25–27]. These results further confirm the reliability and accuracy of the selected pathogenic genes.

### Identification of potential candidate components in HGWD

**Fig. 2:**
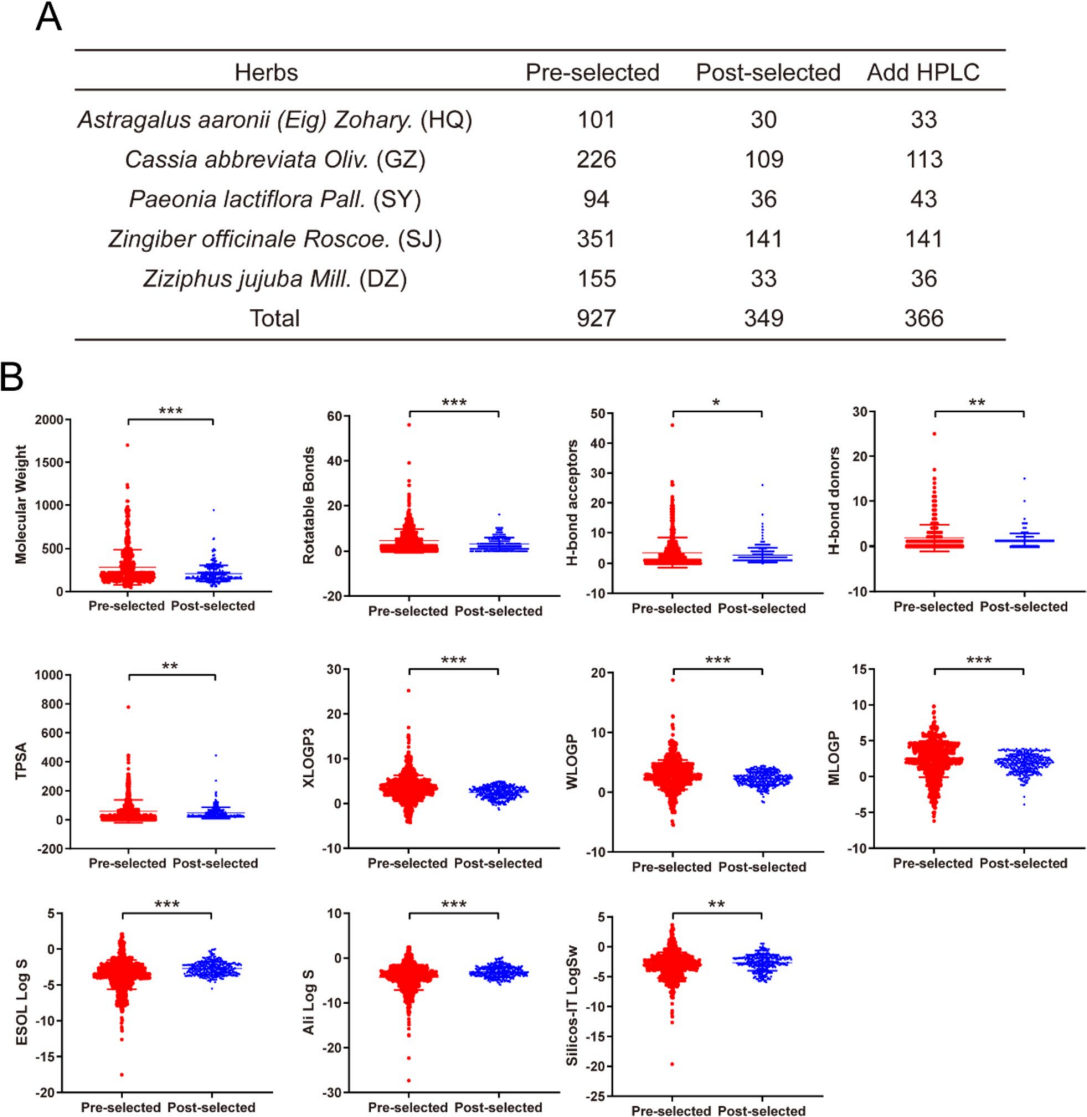
Scatter plots of properties changes between two sets of components. **A** The number of components before and after screening. **B** The red dots represent the componments before ADMET filtering (Pre-selected), and the blue dots represent the componments after ADMET filtering (Post-selected)

A total of 927 components in HGWD were collected from the online databases TCMSP, TCMID, TCM@Taiwan, and YaTCM (Supplementary Table S2). According to the screening criteria, 349 potentially candidate components, including 30 in HQ, 109 in GZ, 36 in SY, 141 in SJ, and 33 in DZ, were retained for further analysis (Fig. 2A, Supplementary Table S2). In addition, compounds with high chemical concentrations in HGWD that typically elicit biological activities were extracted from the literature (Table 1) and combined with 349 potentially candidate components, ultimately yielding 366 candidate components for a more comprehensive evaluation (Fig. 2A, Supplementary Table S3). The changes in properties between pre- and post-selection indicated that candidate components were more consistent with the drug screening criteria and may have better activities (Fig. 2B).

**Table 1:** The candidate components of HGWD collected from literature

Formula	Method	Component	Concentration	Reference
Huangqi Guizhi Wuwu Decoction (HGWD)	UFLC	Calycosin-7-O-β-dglucoside	0.203 μg/mL	[28]
		Paeoniflorin	21.67 μg/mL	
		Albiflorin	4.54 μg/mL	
		Cinnamic acid	0.644 μg/mL	
*Astragalus aaronii* (Eig) Zohary. (Huang Qi, HQ)	HPLC	Campanulin	0.42 mg/g	[29]
		Formononetin	0.02 mg/g	
*Cassia abbreviata* Oliv. (Gui Zhi, GZ)	UHPLC	Protocatechuic acid	0.11 mg/g	[30]
		Coumarin	0.84 mg/g	
		Cinnamic alcohol	0.04 mg/g	
		Cinnamic acid	0.68 mg/g	
		Cinnamaldehyde	9.93 mg/g	
*Paeonia lactiflora* Pall. (Shao Yao, SY)	HPLC	Gallic acid	2.33 mg/g	[31]
		Hydroxyl-paeoniflorin	1.89 mg/g	
		Catechin	0.03 mg/g	
		Albiflorin	4.44 mg/g	
		Paeoniflorin	4.81 mg/g	
		Benzoic acid	0.03 mg/g	
		1,2,3,4,6-pentagalloylglucose	4.80 mg/g	
		Benzoyl—paeoniflorin	0.11 mg/g	
		Paeonol	0.07 mg/g	
*Zingiber officinale* Roscoe (Sheng Jiang, SJ)	HPLC	6-Gingerol	16.62 mg/g	[32]
		6-Shogaol	4.92 mg/g	
*Ziziphus jujuba* Mill. (Da Zao, DZ)	HPLC	Rutin	0.21 mg/g	[33]
		Quercetin	0.008 mg/g	
		Isorhamnetin	0.17 mg/g	

### Analysis of active components and targets

**Fig. 3:**
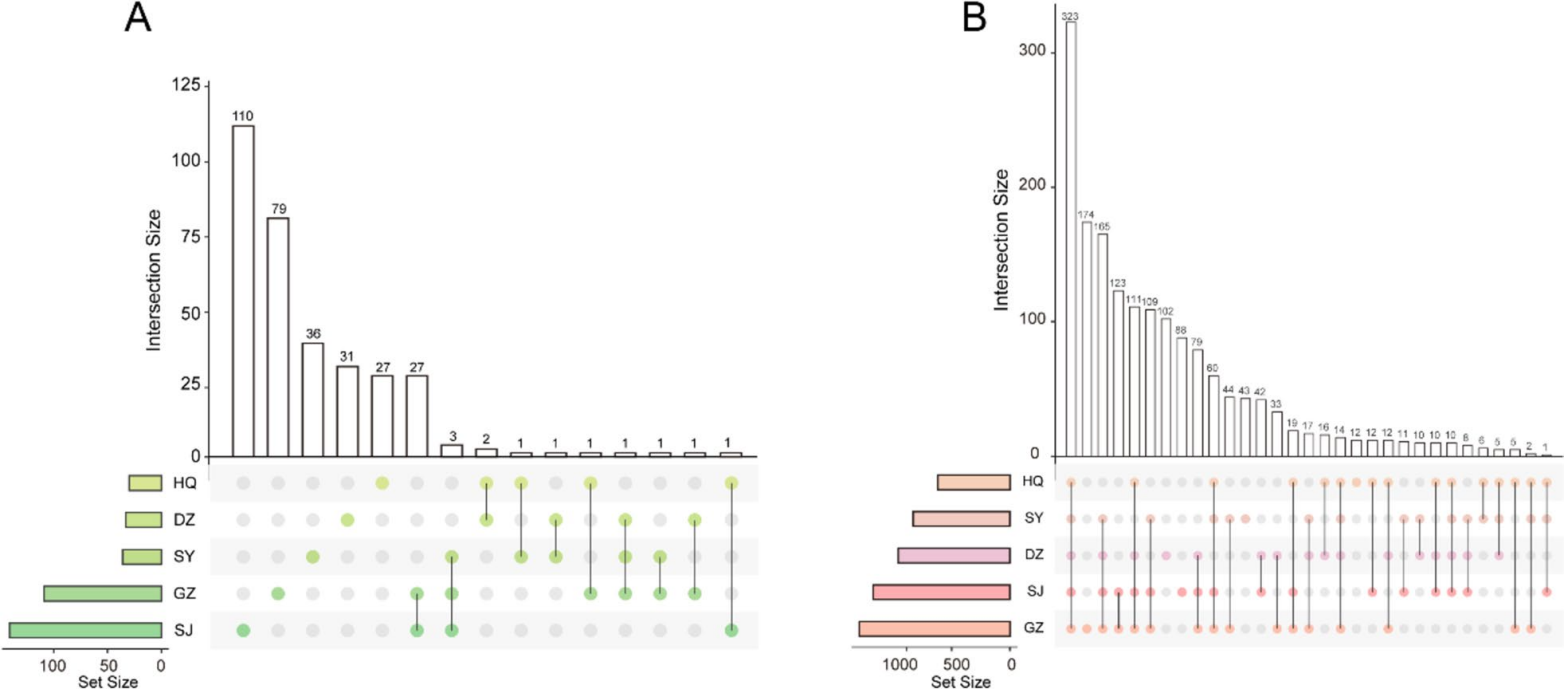
Analysis of candidate components and targets. **A** Shared and specific candidate components of HQ, GZ, SY, GZ and SJ in HGWD. **B** Shared and specific targets of HQ, GZ, SY, GZ and SJ in HGWD

By analyzing the distribution of these candidate components in the five herbs, we found that 39 candidate components co-existed in two or more herbs (Fig. 3A). For example, dibutyl phthalate (MID010507), cedrol (MID010700), and acetic acid (MID014218) are present in GZ, SY, and SJ, respectively. Among these, cedrol has been reported to ameliorates RA by reducing inflammation and selectively inhibiting JAK3 Phosphorylation [34]. Furthermore, GZ, SY, and DZ all contain the component ( +)-catechin (MID010331), which has been reported to synergistically mediate anti-inflammatory effects with quercetin by inhibiting the NF-κB and MAPK pathways [35]. Additionally, GZ and SJ share 27 common components. Among them, thymol (MID011844) was demonstrated to have anti-inflammatory and wound healing-promoting effect [36]; eugenol (MID010096) and ()-Bornyl acetate (MID011038) were reported to be able to mitigate the progression of inflammation [37, 38]. However, apart from the shared components, each herb has its own specific ingredients that perform unique functions. SJ, GZ, SY, DZ, and HQ contained 110, 79, 36, 31, and 27 specific candidate components, respectively. To further explore potential therapeutic mechanisms, we predicted the targets of these five herbs. By integrating information from the SEA, HitPick and Swiss Target online tools, 618 HQ, 1290 targets for GZ, 828 targets SY targets, 1171 SJ targets, and 957 DZ targets were obtained (Supplementary Table S4). The distribution map suggested that 323 targets co-exist in five herbs, and most of them had critical roles in the evolution and intervention of RA (Fig. 3B, Supplementary Table S5). For instance, the activation of the STAT1 pathway has been significantly observed in RA, and inhibiting this pathway can alleviate the progression of the disease [39]. Similarly, the levels of the chemokine CXCL12 were elevated in the synovium and bone tissue of RA patients, and it has a pronounced activating effect on mature osteoclasts by inducing bone resorption activity and specific MMP-9 enzyme release [40]. Furthermore, recent studies have highlighted the importance of synovial hypoxia as a contributing factor in the development of RA, with the involvement of the hypoxia-inducible factor HIF1A being significant in this context [41, 42]. Additionally, MIF has been suggested as a therapeutic target for RA [43], while JAK inhibitors appear to be an important treatment option for RA patients who are difficult to treat [44]. Similarly, each component had its own unique targets. Specifically, HQ, SY, DZ, SJ, and GZ have 12, 43, 102, 88, and 174 targets, respectively, which fully reflects the complex multi-component-target features and synergistic therapeutic mechanisms of TCM formulas. Overall, our data suggest that HGWD can potentially treat RA via coordination of shared and unique components and targets.

### Construction of core functional space and selection of effective proteins

**Fig. 4:**
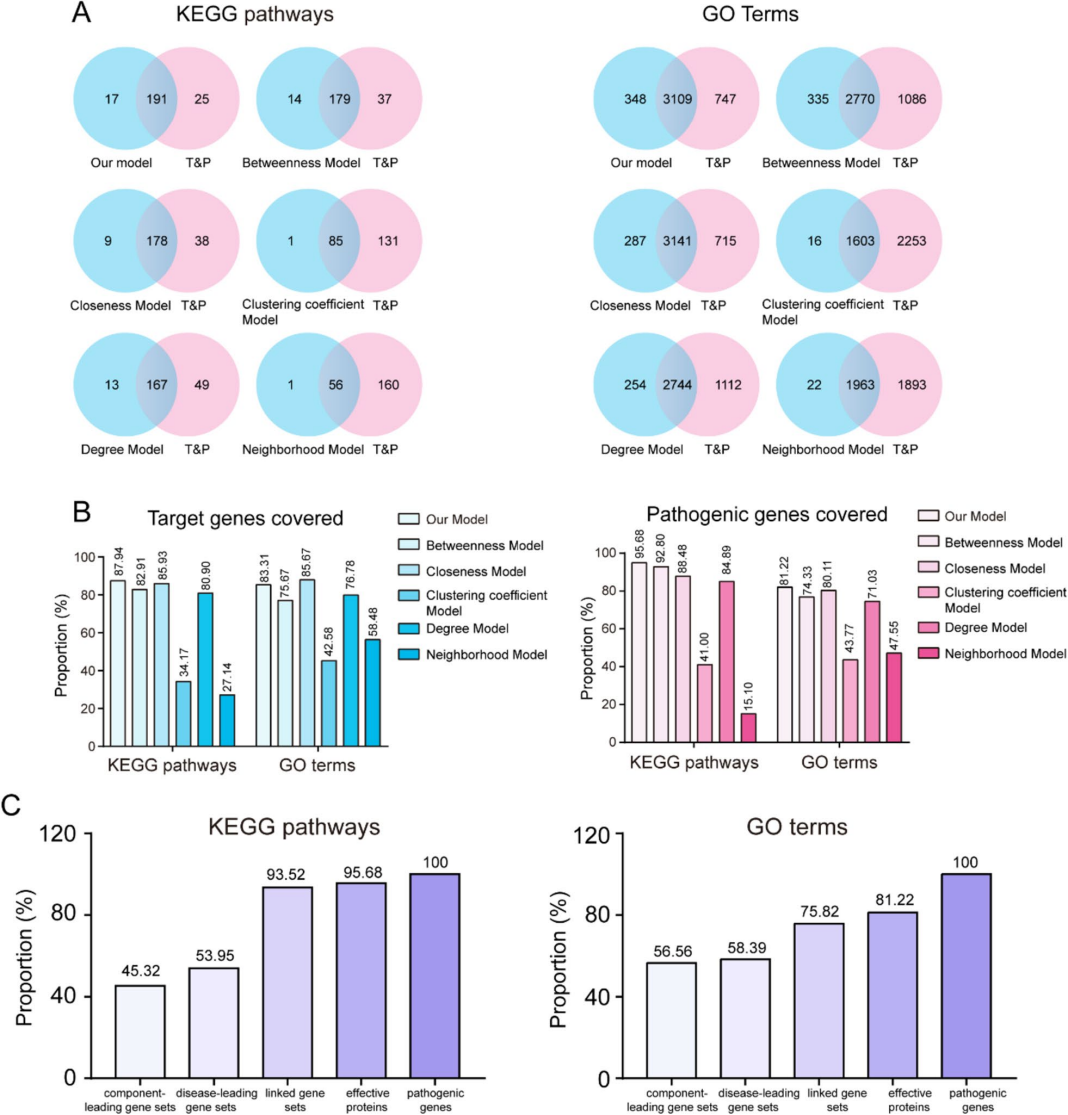
Construction of CFS and selection of effective proteins. **A** Venn diagrams show the number of overlapped KEGG pathways and GO terms between our model and other widely used model. **B** The proportion histogram of KEGG and GO of all model compared with the target genes or pathogenic genes. (C) The proportion histogram of KEGG pathways and GO terms of linked gene sets, disease-leading gene sets, component-leading gene sets and effective proteins compared with pathogenic genes

To further explore the potential therapeutic mechanism of HGWD in RA, 1666 targets after removing duplicate genes (Supplementary Table S6) were used to construct the component-target network (C-T network). However, considering that the C-T network can only reflect some therapeutic effects but cannot reflect the propagation mode of such therapeutic effects, we further integrated pathogenic gene–gene interactions and the C-T network to construct a comprehensive C-T-P network, which could reveal the complex regulatory relationship of organism life activities to a certain extent. Our results showed that the C-T-P network contained 2,495 nodes and 57,683 edges. In C-T-P networks, the node influence is one of the most critical topological attributes. It is generally believed that nodes with an influence score higher than the average node influence score of the C-T-P network are key players and central hubs [45]. In view of this principle, we propose a novel model that considers both the node control force and node bridging force to calculate the influence of nodes in this C-T-P network and reserved these larger-than-average nodes and their edges to construct the core functional space (CFS), thus identifying 796 effective proteins. To verify that the effective proteins obtained from CFS can reflect RA characteristics at the functional level, we performed KEGG and GO analyses and compared them with the target and pathogenic genes (T&P genes). Our results showed that among 216 KEGG pathways and 3856 GO terms enriched in T&P genes, our model covered 191 KEGG pathways and 3109 GO terms with coverage rates of 88.43% and 80.63%, respectively (Fig. 4A). Most of these KEGG pathways and GO terms have been well-documented, with a tight connection to RA. These include PI3K/AKT, MAPK, TNF, and NF-kappa B pathways. In addition, we compared it with other models that are widely used to calculate the influence of nodes, including betweenness centrality, closeness centrality, clustering coefficient, degree, and neighborhood connectivity. The effective proteins obtained from each model were used to perform functional analysis, and their KEGG pathways and GO term coverage with target genes and pathogenic genes were calculated. Our results suggest that our model has a higher percentage of coverage of KEGG pathways and GO terms than the other models, whether compared with T&P genes or target genes and pathogenic genes, respectively (Fig. 4A-B). In conclusion, our model has higher accuracy and reliability for determining critical genes with intervention potential at the functional level.

Three datasets are used in this study. The first type represents genes that directly link the component targets and pathogenic genes, which we describe as linked gene sets. The second type refers to genes that uniquely belong to pathogenic genes, which we defined as disease-leading gene sets. The third type represents genes that specifically target component targets, which were determined to be component-leading gene sets. To evaluate whether the effective proteins screened by CFS could better reflect pathogenic genes, we conducted a functional analysis of the three types. As seen in Fig. 4C, for the KEGG pathway, linked gene sets, disease-leading gene sets and component-leading gene sets accounted for 45.32%, 53.95%, and 93.52% of the pathogenic genes, respectively; However, effective proteins accounted for 95.68%, which was higher than the previous three types. Similarly, for the GO analysis results, linked gene sets, disease-leading gene sets, and component-leading gene sets accounted for 56.56%, 58.39%, and 75.82%, respectively, which were far less than 81.22% of the effective proteins (Fig. 4C). These results provide further validation of the reliability of the model we constructed and verify that effective proteins screened by CFS could represent the function of the pathogenic genes of RA and may play a vital role in the HGWD treatment of RA.

### Identification and validation of key response components group

**Fig. 5:**
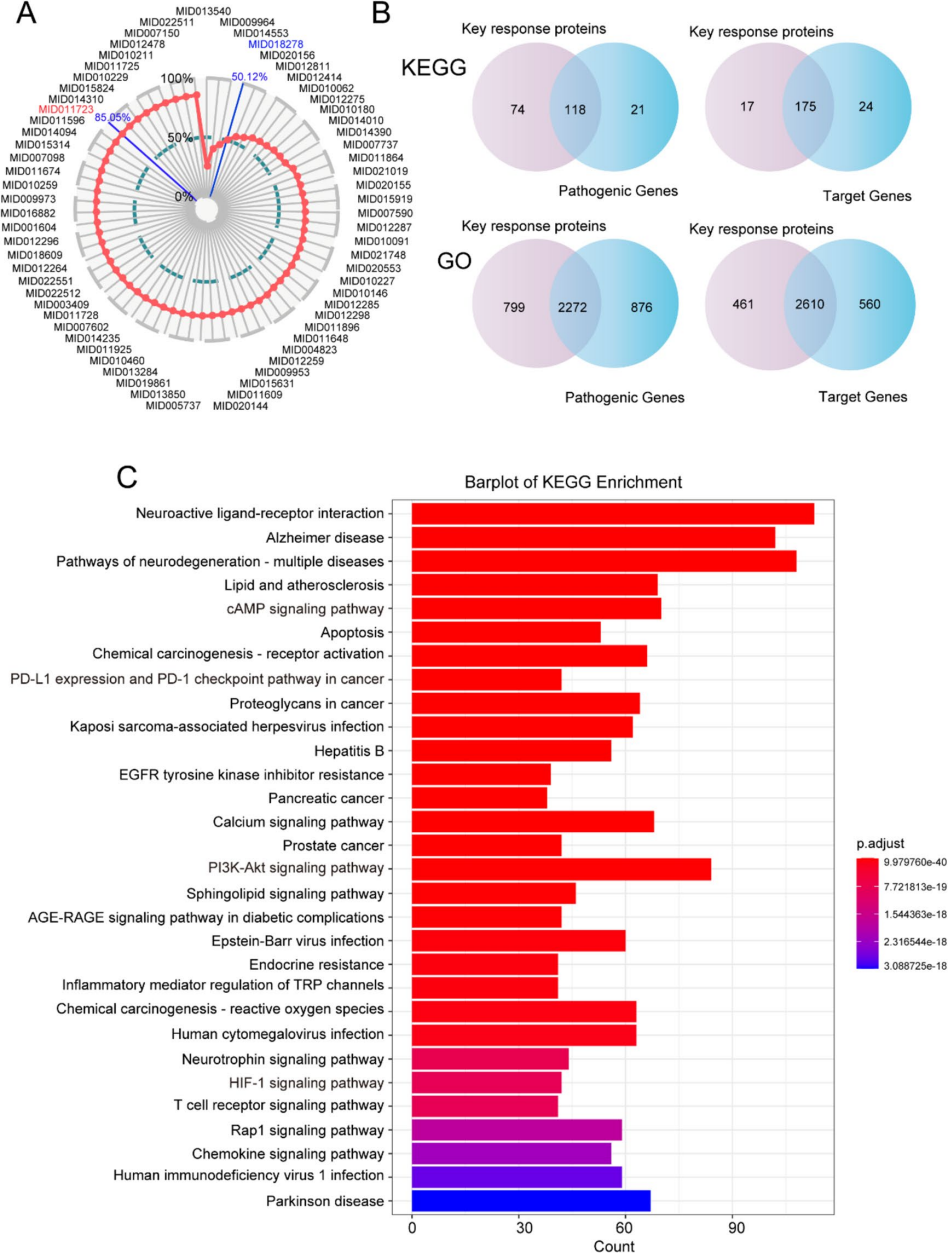
Identification and validation of key response components group. **A** The ICC scores of HGWD active components. **B** The number of overlapped KEGG pathways and GO terms between key response proteins and pathogenic genes. **C** Top 30 KEGG pathways of key response proteins

To further optimize the active components and obtain the key response components group (KRCG), the increasing coverage coefficient (ICC) model was orchestrated for evaluating cumulative contribution rate of each component. Based on the output of the cumulative contribution rate score, only the first four components, benzyl acetate (MID013540), vanillic acid (MID009964), butyl benzoate (MID014553), and moupinamide (MID018278), accounted for 50.12% of the effective proteins. Furthermore, 59 components with 677 targets contributed to 85.05% target coverage, defined as KRCG (Fig. 5A; Supplementary Table S7).

To further evaluate the effectiveness of KRCG in treating RA at the functional level, we extracted 677 targets produced by KRCG as key response proteins and compared them with pathogenic genes and target genes at the KEGG pathway and GO term levels. Our results showed that key response proteins enriched 192 KEGG pathways, accounting for 84.89% and 87.94% of the pathogenic genes and target genes, respectively, and that 3071 GO terms were enriched with key response proteins, accounting for 72.17% and 82.33% of the pathogenic genes and target genes, respectively (Fig. 5B). These data demonstrate that the proposed model effectively selected key targets and eliminated noise. Figure 5C shows the top 30 signaling pathways analyzed by KRCG enrichment, mainly involving cAMP, EGFR tyrosine kinase inhibitor resistance, calcium signaling, PI3K-Akt, and HIF-1 pathways. Among these, the cAMP, PI3K-Akt, and HIF-1 pathways have been extensively associated with the progression or treatment of RA. In summary, KRCG screening partially revealed the underlying mechanism of HGWD treatment for RA at the functional level.

### Verification of KRCG in vitro

**Fig. 6:**
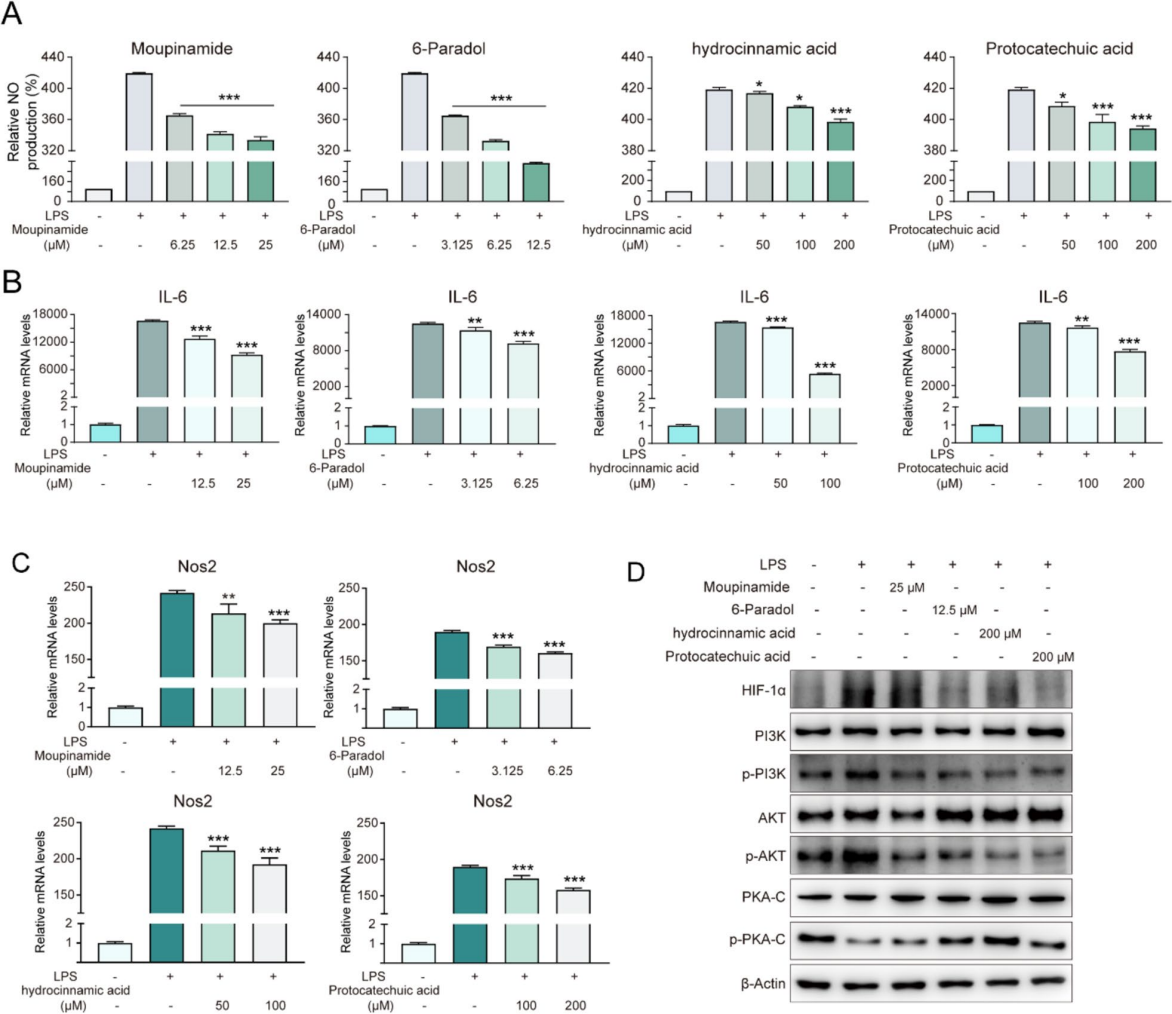
Verification of KRCG in vitro. **A** Cells were treated with moupinamide, 6-Paradol, hydrocinnamic acid and protocatechuic acid at the indicated concentrations for 24 h and then the NO production was measured. **B**-**C** The IL-6 and Nos2 mRNA levels were detected by qPCR. **D** The protein expression levels were determined by western blot (Full-length blots/gels are presented in Supplementary Figure S2)

We randomly selected four components (moupinamide, MID018278; 6-Paradol, MID012414; hydrocinnamic acid; MID010180, and protocatechuic acid; MID007590) to verify the effectiveness of KRCG and the reliability of our selection model at the experimental level. The cell viability of four components on RAW 264.7 cells were measured by CCK-8 and the results showed that the components were not significantly cytotoxicity to RAW264.7 cells at concentrations below 25 μM for moupinamide, 12.5 μM for 6-Paradol, 200 μM for hydrocinnamic acid and 200 μM for protocatechuic acid (Supplementary Figure S1). Therefore, these concentration ranges without cytotoxic effects were used in subsequent experiments. The pathogenesis of RA is usually manifested by an increase in interleukin 6 (IL-6), Nos2, nitric oxide (NO), and other inflammatory factors as well as activation of the MAPK and NF-κB pathways. Hence, the NO production level was determined, and the results showed a 4.2-fold increase in the LPS model group compared to the control group, indicating that LPS successfully triggered an inflammatory response in RAW264.7 cells. However, when treated with moupinamide (6.25, 12.5 and 25 μM), 6-Paradol (3.125, 6.25 and 12.5 μM), hydrocinnamic acid (50, 100 and 200 μM) and protocatechuic acid (50, 100 and 200 μM), the release of NO has significantly decreased in a concentration-dependent manner (Fig. 6A), which indicated the anti-inflammatory effects of the four components. To further verify their anti-inflammatory effects, we measured intracellular levels of Nos2 and IL-6, which are typical markers of the inflammatory response. As shown in Fig. 6B and 6C, the levels of Nos2 and IL-6 were markedly increased in the LPS model group, whereas they were reduced in a dose-dependent manner in the administration group (Fig. 6B-C). Overall, our results demonstrated that all four components effectively inhibited the LPS-induced inflammatory response in RAW264.7 cells, further confirming the effectiveness of KRCG.

Similarly, we conducted experimental validation on three randomly selected non-KRCG components. The CCK8 results demonstrated that concentrations below 200 uM for nerol, and below 400 uM for geranyl acetate and bifendate did not exhibit any significant toxic effects on RAW264.7 cells (Supplementary Figure S3A). Furthermore, the relative NO production results indicated that none of the tthree components had a mitigating effect on the LPS-induced inflammatory response in RAW264.7 cells (Supplementary Figure S3B). Importantly, there were no statistically significant differences observed in the levels of IL-6 and Nos2 mRNA (Supplementary Figure S3C). These findings provide further evidence of the significance of KRCG in the treatment of RA and validate the reliability of our new model.

Moreover, to reveal the potential underlying mechanism of KRCG, western blotting was performed to detect signaling pathways enriched in key response proteins (Fig. 5C), including cAMP, PI3K-Akt, and HIF-1 pathways. The data showed that the PI3K-Akt and HIF-1 pathways were activated and the cAMP pathway was inhibited in the LPS model group. However, these pathways were partially rescued by the treatment with these four components. For example, 25 μM moupinamide inhibited LPS-induced activation of p-PI3K and p-AKT but had no significant effect on the cAMP and HIF-1α pathways. However, Paradol at 12.5 μM, hydrocinnamic acid at 200 μM and protocatechuic acid at 200 μM both significantly inhibited the activation of PI3K-Akt pathway and HIF-1 pathway as well as promoting the activation of cAMP pathway (Fig. 6D). In summary, our results verified the reliability of KRCG and demonstrated its potential synergistic functional role in RA therapy.

## Discussion

In this study, we aimed to investigate the core components and mechanisms of HGWD in the treatment of RA. we developed an optimized model for analyzing the correlation between disease-related genes and the targets of TCM components. This model incorporated both node control force and node bridging force to construct the CRS, which was further used to identify the KRCG through an ICC model. The results identified 59 KRCG of HGWD in treating RA and further revealed their potential pharmacological mechanisms for inhibiting the inflammatory response by synergistically targeting the cAMP, PI3K-Akt, and HIF-1α pathways.

In fact, there are multiple computational models available to assess the centrality and importance of nodes in a network. These models include betweenness centrality, closeness centrality, clustering coefficient, degree centrality, and neighborhood connectivity. Each of these models has distinct focuses and calculation methods when measuring node importance. Betweenness centrality quantifies the criticality of a node in information flow by measuring its frequency of occurrence in all shortest paths [46, 47]. Closeness centrality measures the average distance between a node and other nodes in the network [48]. The clustering coefficient gauges the level of connectivity among a node’s neighboring nodes [49]. Degree centrality, being the simplest measure of node importance, evaluates the number of connections a node has with other nodes [50]. Neighborhood connectivity measures the degree of connectivity among a node’s neighboring nodes [51]. In our study, we propose a novel computational model that integrates node control force and node bridging force. This model exhibits the highest score when computing the KRCG of HGWD compared to other computational models. It effectively retains the active components responsible for the functionality of HGWD, as evidenced by the KEGG and GO enrichment results shown in Fig. 4B. Additionally, recent research utilizing ultra-performance liquid chromatography-diode array detector (UPLC-DAD) fingerprint identified five compounds in HGWD with a relative content > 1%: paeoniflorin, astragaloside IV, hexahydrocurcumin, formononetin, and calycosin-7-glucoside. Experimental studies have demonstrated the anti-inflammatory effects of these compounds [52]. Notably, paeoniflorin, hexahydrocurcumin, and formononetin are all present in the KRCG, while the other two components were not initially included in the component survey. This finding validates the reliability and trustworthiness of our model, while also highlighting a significant limitation of computational models: the dependence of prediction results on the quality and reliability of input data, which may be compromised by errors or missing data. Furthermore, our experimental validation focused on assessing the anti-inflammatory effects of four components: moupinamide, 6-Paradol, hydrocinnamic acid, and protocatechuic acid. The findings revealed their synergistic targeting of the cAMP, PI3K-Akt, and HIF-1α pathways, aligning with previous literature reports [53, 54].

However, our study has several limitations that should be acknowledged. Firstly, the predictive outcomes of computational models heavily rely on the completeness and reliability of input data. Unfortunately, we were unable to collect comprehensive information regarding the components and disease targets of the HGWD. Secondly, the lack of extensive validation experiments poses a limitation that needs to be addressed. It is crucial to validate the reliability of our model by incorporating a wider range of components from HGWD. Additionally, evaluating the individual effectiveness of KRCG in comparison to the HGWD is necessary. Furthermore, the identification of effective components in HGWD using HPLC is of utmost importance, as their contribution to therapeutic effects largely depends on their concentration levels. Lastly, while our study primarily focuses on unveiling the role of KRCG, the significance of non-KRCG should not be disregarded, necessitating further research to elucidate the synergistic effects of the entire formulation.

Therefore, a comprehensive approach encompassing qualitative and quantitative analysis of TCM using HPLC, ensuring data integrity through the incorporation of information from databases, as well as the utilization of computational models and extensive experimental validation, will significantly enhance the accuracy and reliability of computational models, and deepen the overall understanding and application of TCM.

### Supplementary Information

**Additional file 1.**

**Additional file 2.**

**Additional file 3.**

**Additional file 4.**

**Additional file 5.**

**Additional file 6.**

**Additional file 7.**

**Additional file 8: Figure S1. **The toxicity of moupinamide, 6-Paradol, hydrocinnamic acid, and protocatechuic acid were evaluated by CCK8.

**Additional file 9: Figure S2.** The full-length blots of Hif-1α (A) (The six lanes on the right), PI3K (B), p-PI3K (C), AKT (D), p-AKT (E), PKA C (F) (The six lanes on the left), p-PKA C (G) and β-actin (H).

**Additional file 10: Figure S3. **(A)The toxicity of geranyl acetate, nerol, and bifendate were evaluated by CCK8. (B) NO production was detected after the cells were treated with the indicated concentration for 24 h. (C) The IL-6 and Nos2 mRNA levels were detected by qPCR. * 0.01 < *P* < 0.05, ** 0.001 <* P* < 0.01, and *** *P* < 0.001. n.s., no significance.

## Data Availability

The link to the GEO dataset used in the article is as follows: https://www.ncbi.nlm.nih.gov/geo/query/acc.cgi?acc=GSE55235. The All data related to this study are included in the article/supplementary material and further inquiries can be directed to the corresponding author.
