# Supplementary material for: Screening of the key response component groups and mechanism verification of Huangqi-Guizhi-Wuwu-Decoction in treating rheumatoid arthritis based on a novel computational pharmacological model
Source: BMC Complement Med Ther. 2024 Jan 2;24:4. doi: 10.1186/s12906-023-04315-y (PMC10759359; doi:10.1186/s12906-023-04315-y)
Supplement: Supplementary file 8 — Additional file 8: Figure S1. The toxicity of moupinamide, 6-Paradol, hydrocinnamic acid, and protocatechuic acid were evaluated by CCK8. [file 12906_2023_4315_MOESM8_ESM.docx]

Supplementary Materials for

Uncovering the pharmacological mechanism of Huangqi-Guizhi-Wuwu-Decoction in treating rheumatoid arthritis via integrative pharmacology and experimental validation

Qinwen Liu ^1,2#^, Qian Luo ^1,2#^, Qiling Fan ^1,2#^, Yi Li^1,2^, Aiping Lu^3,4*^, Daogang Guan^1,2*^


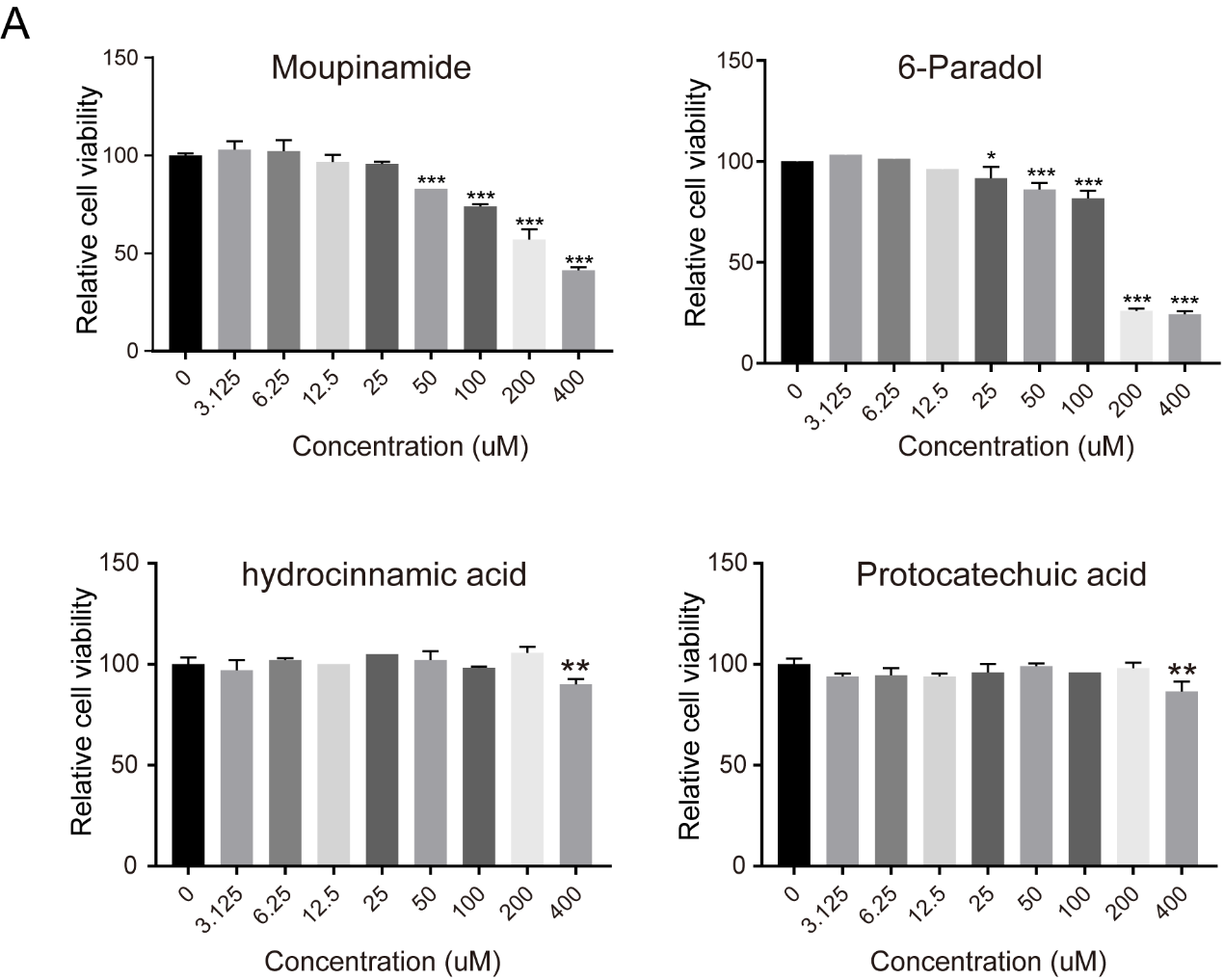


**Figure. S1.** The toxicity of moupinamide, 6-Paradol, hydrocinnamic acid, and protocatechuic acid were evaluated by CCK8.
