# Supplementary material for: Screening of the key response component groups and mechanism verification of Huangqi-Guizhi-Wuwu-Decoction in treating rheumatoid arthritis based on a novel computational pharmacological model
Source: BMC Complement Med Ther. 2024 Jan 2;24:4. doi: 10.1186/s12906-023-04315-y (PMC10759359; doi:10.1186/s12906-023-04315-y)
Supplement: Supplementary file 9 — Additional file 9: Figure S2. The full-length blots of Hif-1α (A) (The six lanes on the right), PI3K (B), p-PI3K (C), AKT (D), p-AKT (E), PKA C (F) (The six lanes on the left), p-PKA C (G) and β-actin (H). [file 12906_2023_4315_MOESM9_ESM.docx]

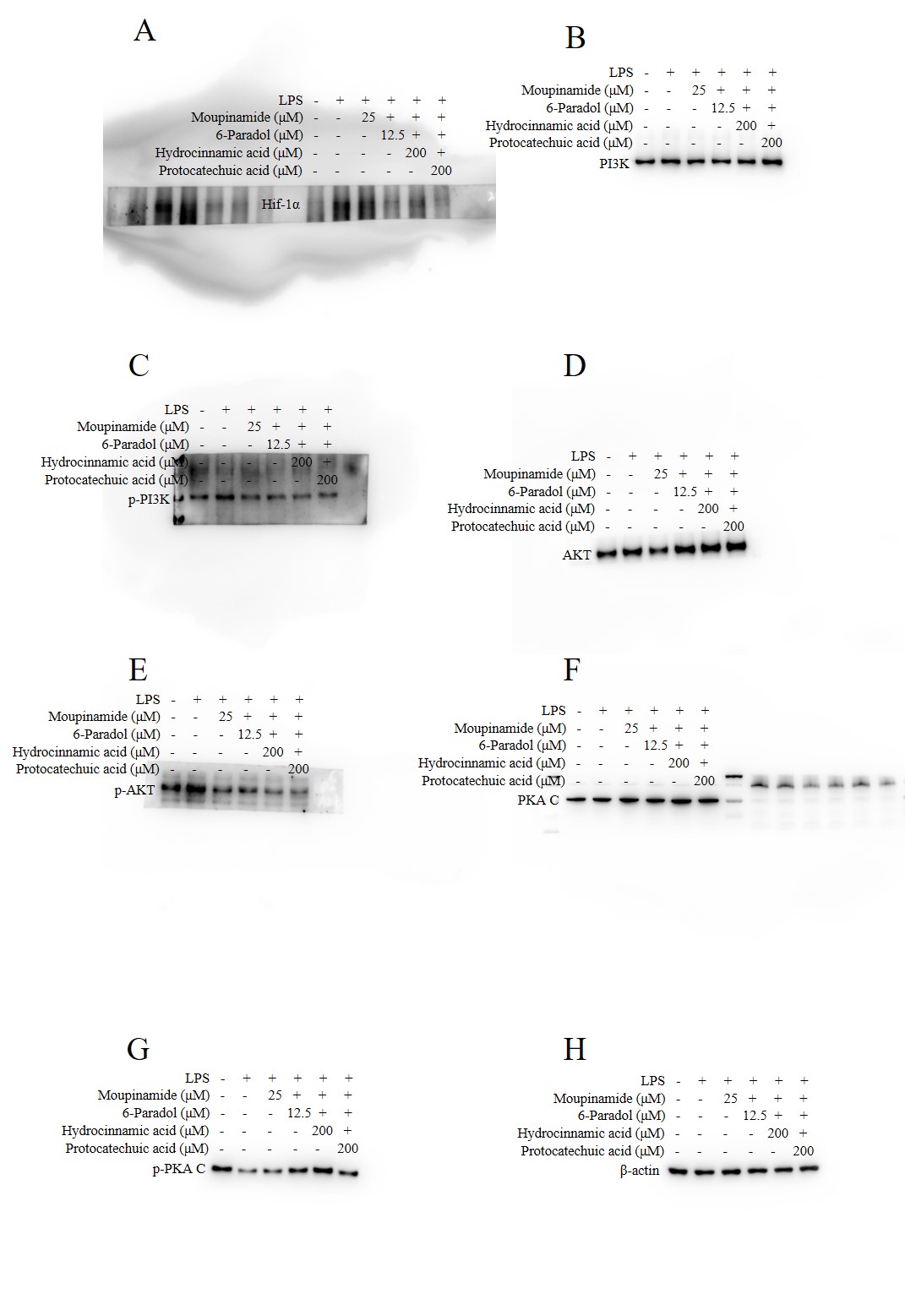


**Figure. S2.** The full-length blots of Hif-1α (A) (The six lanes on the right), PI3K (B), p-PI3K (C), AKT (D), p-AKT (E), PKA C (F) (The six lanes on the left), p-PKA C (G) and β-actin (H).
