# Supplementary material for: Screening of the key response component groups and mechanism verification of Huangqi-Guizhi-Wuwu-Decoction in treating rheumatoid arthritis based on a novel computational pharmacological model
Source: BMC Complement Med Ther. 2024 Jan 2;24:4. doi: 10.1186/s12906-023-04315-y (PMC10759359; doi:10.1186/s12906-023-04315-y)
Supplement: Supplementary file 10 — Additional file 10: Figure S3. (A)The toxicity of geranyl acetate, nerol, and bifendate were evaluated by CCK8. (B) NO production was detected after the cells were treated with the indicated concentration for 24 h. (C) The IL-6 and Nos2 mRNA levels were detected by qPCR. * 0.01 < P < 0.05, ** 0.001 < P < 0.01, and *** P < 0.001. n.s., no significance. [file 12906_2023_4315_MOESM10_ESM.docx]

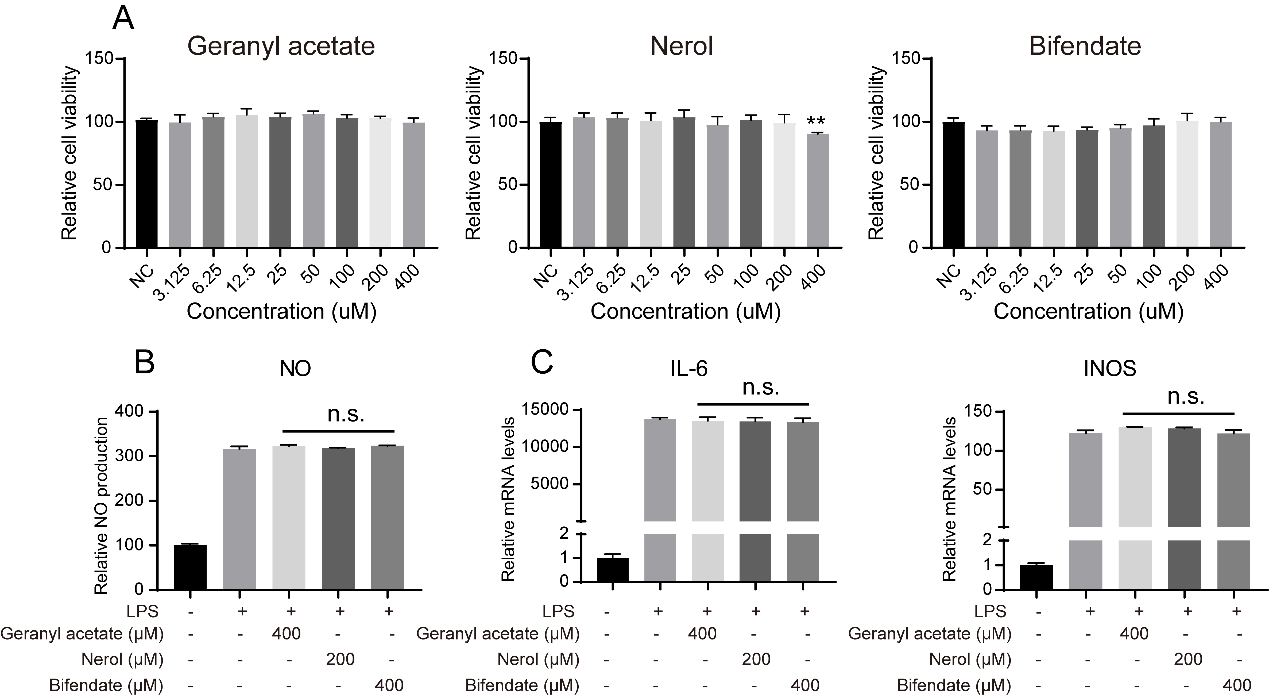


**Figure. S3.** (A)The toxicity of geranyl acetate, nerol, and bifendate were evaluated by CCK8. (B) NO production was detected after the cells were treated with the indicated concentration for 24 h. (C) The IL-6 and Nos2 mRNA levels were detected by qPCR. * 0.01 < *P* < 0.05, ** 0.001 < *P* < 0.01, and *** *P* < 0.001. n.s., no significance
